# Trans-mitochondrial coordination of cristae at regulated membrane junctions

**DOI:** 10.1038/ncomms7259

**Published:** 2015-02-17

**Authors:** Martin Picard, Meagan J. McManus, György Csordás, Péter Várnai, Gerald W. Dorn II, Dewight Williams, György Hajnóczky, Douglas C. Wallace

**Affiliations:** 1 Center for Mitochondrial and Epigenomic Medicine, The Children’s Hospital of Philadelphia and University of Pennsylvania, Philadelphia, Pennsylvania 19104, USA; 2 MitoCare Center, Department of Pathology, Anatomy and Cell Biology, Thomas Jefferson University, Philadelphia, Pennsylvania 19107, USA; 3 Department of Physiology, Semmelweis University, P.O. Box 259, Budapest H-1444, Hungary; 4 Center for Pharmacogenomics, Department of Internal Medicine, Washington University School of Medicine, St Louis, Missouri 63110, USA; 5 Penn EM Resource Laboratory, University of Pennsylvania, Philadelphia, Pennsylvania 19104, USA

## Abstract

Reminiscent of bacterial quorum sensing, mammalian mitochondria participate in inter-organelle communication. However, physical structures that enhance or enable interactions between mitochondria have not been defined. Here we report that adjacent mitochondria exhibit coordination of inner mitochondrial membrane cristae at inter-mitochondrial junctions (IMJs). These electron-dense structures are conserved across species, resistant to genetic disruption of cristae organization, dynamically modulated by mitochondrial bioenergetics, independent of known inter-mitochondrial tethering proteins mitofusins and rapidly induced by the stable rapprochement of organelles via inducible synthetic linker technology. At the associated junctions, the cristae of adjacent mitochondria form parallel arrays perpendicular to the IMJ, consistent with a role in electrochemical coupling. These IMJs and associated cristae arrays may provide the structural basis to enhance the propagation of intracellular bioenergetic and apoptotic waves through mitochondrial networks within cells.

## Abstract (web summary)

Mammalian mitochondria are capable of inter-organelle communication, but connections between mitochondria have not been defined. Here, Picard *et al*. report the presence of inter-mitochondrial junctions, electron-dense regions with coordinated inner membrane cristae that do not depend on mitofusins for their formation.

## Graphical abstract

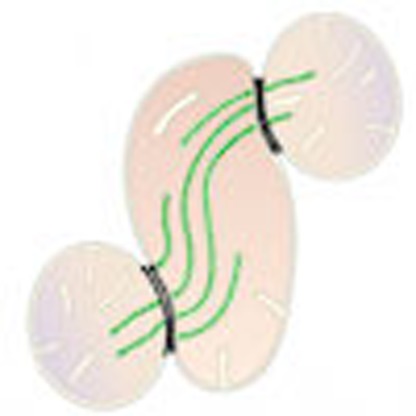

In recent decades, the concept of static, oblong-shaped mitochondria has evolved to a dynamic model where mitochondria behave within mammalian cells as a physically and functionally interconnected network of organelles. Mitochondrial networking, with other organelles^1^ and with each other, occurs in part via dynamic fusion and fission processes, disorders of which are recognized causes of human disease^2^. Thus, connectivity and communication of mitochondria within the cytoplasm appear crucial to cellular homeostasis^3^,^4^. Such networking is reminiscent of the behaviour of mitochondria’s ancestor, the bacteria^5^, which exchange signals enabling quorum sensing, leading to synchronization of gene expression and unified community action^6^. However, structural elements that could enable or facilitate similar interactions among mitochondria within eukaryotic cells have not been established.

In particular, mitochondrial communication involves inter-mitochondrial transmission of the electrochemical gradient or electrochemical coupling^7^,^8^,^9^. Apoptotic signalling involving mitochondrial depolarization also proceeds in waves across the mitochondrial network^10^,^11^. Importantly, such rapid events of inter-mitochondrial transmission travel further than do intra-mitochondrial matrix components, distinguishing them from events of complete mitochondrial fusion^7^. Nevertheless, rapid inter-mitochondrial communication involves physical proximity and contact between mitochondria^7^,^12^. This could serve the purpose of equilibrating the energetic state across adjacent mitochondria. Earlier work suggested the presence of inter-mitochondrial junctions (IMJs), which correlated with electrical coupling between adjacent mitochondria^13^.

With this model in mind, we used transmission electron microscopy (TEM) and tomographic reconstructions to investigate sites of physical interaction between mitochondria in various tissues and animal species. We discovered that specialized electron-dense IMJ structures are associated with increased cristae junction numbers and coordination of cristae orientation between mitochondria. Linking mitochondria to one another with an inducible synthetic linker system in living cells rapidly induced IMJs and associated cristae. These results reveal previously unrecognized coordination of mitochondrial ultrastructure at sites of physical interactions, with implications for the transfer of information between organelles.

## Results

### Mitochondrial contacts at IMJs

**Figure 1:**
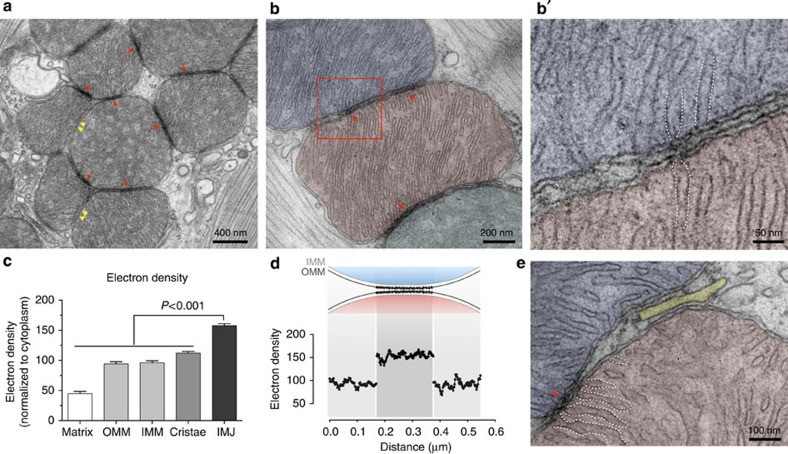
Electron-dense inter-mitochondrial junctions (IMJs) link adjacent mitochondria in the heart. (**a**) Electron micrograph of mouse cardiomyocytes showing mitochondria with electron-dense IMJs (red arrows) and non-electron-dense contacts (yellow double arrowheads). (**b**,**b′**) Higher magnification showing apposed outer mitochondrial membranes and increased membrane electron density. (**c**) Relative electron density of mitochondrial membrane structures (means±s.e.m., one-way analysis of variance with Dunnett’s multiple comparisons, *n*=20 per group). (**d**) Diagram of the relative electron density of IMJ across 20 IMJs. (**e**) IMJs are unique to mito–mito contacts (arrow) and do not form with the sarcoplasmic reticulum (yellow) here juxtaposed to the mitochondrial outer membrane. Cristae membranes forming junctions at IMJs are outlined with dotted lines (also in **b′**).

We began our investigation in mouse heart, the tissue of highest mitochondrial volume density in mammals. At relatively high magnification, the majority of adjacent mitochondria exhibit sites of membrane contact with enhanced electron density, defined as IMJs (Fig. 1a, red arrows). This is consistent with the notion of a mitochondrial syncytium^13^,^14^. However, the simple juxtaposition of organelles does not necessarily produce an IMJ (Fig. 1a, yellow arrowheads), indicating a certain degree of biological regulation, rather than a ubiquitous, obligatory feature of mitochondrial proximity. Higher-magnification TEM at individual membrane resolution reveals that the outer (OMMs) and inner (IMMs) mitochondrial membranes of adjacent mitochondria at IMJs remain distinct (Fig. 1b). Adjacent OMMs were separated by ~7.8±3.7 nm (M±s.d.; Fig. 1b).

At high magnification, apposed IMJ OMMs exhibit enhanced electron density throughout their length (Fig. 1c,d). At the present time, the nature of this enhanced electron density is unknown, but it is likely a result of increased protein and cofactor density. Notably, existing sites of tethering and interaction between mitochondria and the endoplasmic reticulum, plasma membrane and other organelles^1^,^15^ does not show comparable membrane electron density (Fig. 1e). Thus, electron-dense IMJs constitute a phenomenon specific to mitochondria–mitochondria (mito-mito) interactions.

A survey of published images from the literature indicates that IMJs exist across a variety of mammalian species including rodents, human and amphibians, and across tissues including the heart, skeletal muscle, brain, brown fat, retina and glioma (Supplementary Table 1). To determine whether these structures are conserved in other animal species, we examined electron micrographs of the striated muscle from lower organisms including arthropods (flies) and mollusk (scallop; Supplementary Fig. 1). All contain IMJs. The presence of IMJs and associated specific cristae distribution is thus phylogenetically and evolutionary conserved.

### Mitochondrial cristae number is increased at IMJs

The mitochondrial electron transport chain is located within mitochondrial cristae where it generates the electrochemical potential across the invaginated IMM, which is used for ATP synthesis. Cristae membranes form tubular openings as they join with the inner boundary membrane, termed cristae junctions, which are regulated by the ‘cristae organizing system’^16^. At cristae junctions, molecules and ions are exchanged between the cristae invaginations and inter-membrane space^17^,^18^,^19^. In mouse heart and skeletal muscle mitochondria, ultrathin section TEM imaging consistently suggested that cristae junctions were more abundant at IMJs (see Fig. 1e, dotted lines). To examine this, we produced tomograms to quantify cristae junction density per surface area of mitochondrial OMM (Supplementary Fig. 2).

**Figure 2:**
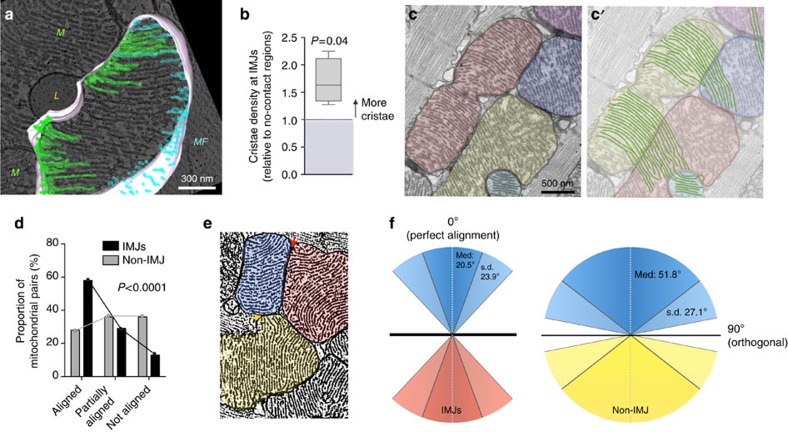
Mitochondrial cristae are more abundant and coordinate at IMJs. (**a**,**b**) Three-dimensional tomographic reconstruction used to quantify cristae abundance at mito–mito (green) and mito–myofibrils (MF, blue) contacts. L, lipid droplet, (mean±s.e.m., paired *T*-test, *n*=510 cristae from four complete tomograms). (**c**,**c′**) Cardiomyocyte mitochondria with cristae exhibiting a high degree of trans-mitochondrial alignment. (**d**) Pairs of mitochondria analyzed for degree of cristae alignment at IMJs and non-electron-dense contacts (non-IMJ) showing preferential alignment at IMJs. (mean±s.e.m., *χ*^2^, *n*=83–151 pairs per group). (**e**) ‘Fingerprint’ electron micrograph of mouse heart cardiomyocyte mitochondria processed to outline cristae, illustrating representative events of mitochondrial alignment at electron-dense junction sites (IMJ, red arrow), and non-alignment at non-electron-dense contacts (non-IMJ, yellow arrow). (**f**) Quantification of incident angles between mitochondria joined by IMJ or non-IMJ. IMJ inter-mitochondrial cristae angles exhibit less variability and closer to exact orientation (angle of 0°) than non-IMJ. Med, median.

Cristae form junctions more frequently at IMJs (+69.7±20.6%, M±s.e.m.) compared with areas of mitochondria facing non-mitochondrial components such as myofibrils (Fig. 2a,b). Cristae arising from junctions at IMJs also tend to have fewer fenestrations (openings in the cristae membrane plane), yielding longer profiles when sectioned longitudinally (Fig. 2a). Among the potential mechanisms to explain this finding, it is notable that protons produce deep cristae-like invaginations within IMM-like cardiolopin-containing membranes^20^. Thus, if protons accumulated preferentially at IMJs, this could account for the predominance of continuous cristae radiating perpendicularly (see below) from the contact sites.

### Cristae of adjacent mitochondria are coordinated at IMJs

That IMJs are involved in mito–mito interactions is further indicated by the observation that cristae membranes exhibit a high degree of alignment between adjacent organelles (see examples in Supplementary Fig. 3). Trans-mitochondrial cristae alignment is most clearly visualized through a series of consecutive frames spanning the thickness of a tomogram (~200 nm) where several mitochondria form IMJs in mouse heart (Supplementary Video 1).

This inter-mitochondrial coordination of cristae in cardiomyocytes often involves multiple organelles organized in clusters (Fig. 2c,c′). When comparing pairs of neighbouring mitochondria at IMJs, 58.3% of pairs have aligned cristae (<30° incident angle) compared with 13.2% non-aligned (60–90°) (*χ*^2^ *P*<0.0001, Fig. 2d). In contrast, cristae at non-IMJ contacts, where electron density is unchanged, are randomly oriented with no evidence of alignment between adjacent mitochondria (Fig. 2d,e). As a result, the median incident angle between mitochondria linked by an IMJ is 20.5±23.9°, compared with 51.8±27.1° for non-IMJ contacts (*χ*^2^ *P*<0.01, Fig. 2f). Thus, cristae orientation, curvature and density per surface area are non-random, exhibiting directionality and orientation that may enable cristae membranes of adjacent mitochondria to become physically and energetically coordinated. Such trans-mitochondrial coordination of cristae at IMJs would account for the collateral accumulation of membrane potential-sensitive dyes in adjacent mitochondria observed in living cultured human cells^21^.

**Figure 3:**
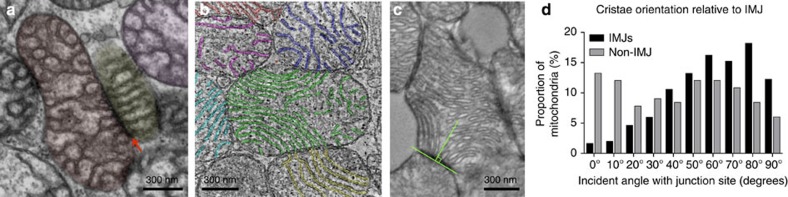
Preferred cristae orientation and organisation at IMJs. Disruption of mitochondrial function and cristae architecture by *Ant1* deletion and *ND6* mutation does not eliminate IMJs (arrow) and trans-mitochondrial cristae alignment in the skeletal muscle (**a**) or the heart (**b**). See Supplementary Video 1 for animation of tomogram (**b**). (**c**) Orientation of cristae relative to the tangent of mito-mito contacts (IMJs and non-IMJ) quantified on electron micrographs. An incident angle of 0° indicates that cristae lie parallel to the site of contact, whereas an angle of 90° indicates perpendicular cristae orientation. (**d**) Frequency distribution of cristae angle in both IMJ and non-IMJ (*P*<0.01). Note the near-random distribution of cristae orientations at non-IMJs. Frequency distributions compared based on 99% confidence interval of the mean, *n*=302 IMJs and 166 non-IMJ contacts.

To determine whether cristae energetics and organization are obligatory for IMJ formation, we experimentally disrupted these properties by genetically ablating the adenine nucleotide translocase 1 (*Ant1*^−/−^)^22^ and introducing a pathogenic mitochondrial DNA (mtDNA) mutation in the NADH dehydrogenase subunit 6 (*ND6*)^23^, which codes for a subunit of the electron transport chain. This deleterious genetic combination render cristae highly disorganized. Yet in this context, trans-mitochondrial cristae alignment still occurs, and mitochondrial IMJs still form (Fig. 3a). Thus, even when mitochondrial cristae are naturally dysmorphic, the physical juxtaposition associated with IMJ leads to cristae alignment across neighbouring mitochondria.

Within normal heart mitochondria, cristae membranes occasionally exhibit a high degree of membrane curvature (see Fig. 3b and Supplementary Fig. 3). This occurs when the cristae within a single mitochondrion extend between the two IMJs formed with different mitochondria. Measuring the incident angle of cristae membranes relative to the tangent of IMJs and non-IMJ contacts (Fig. 3c) reveals that the presence or absence of IMJs influence cristae orientation. Whereas cristae emerging from non-electron-dense contacts have no preferred incident angle, cristae at IMJs are preferentially arrayed at right angles from the contact site (Fig. 3d). The distribution of cristae angles at non-IMJ is near random (skewness=0.02), but the distribution at IMJs is negatively skewed (skewness=−0.61) with significantly fewer low angles at IMJs than expected by chance (Fig. 3d). To maintain cristae continuity in this manner, being constrained at the two ends at IMJs, the cristae membranes must occasionally bend. Such lipid bilayer conformation is energetically unfavourable, implying some degree of regulation and functional relevance^24^.

### IMJs are physiologically regulated

**Figure 4:**
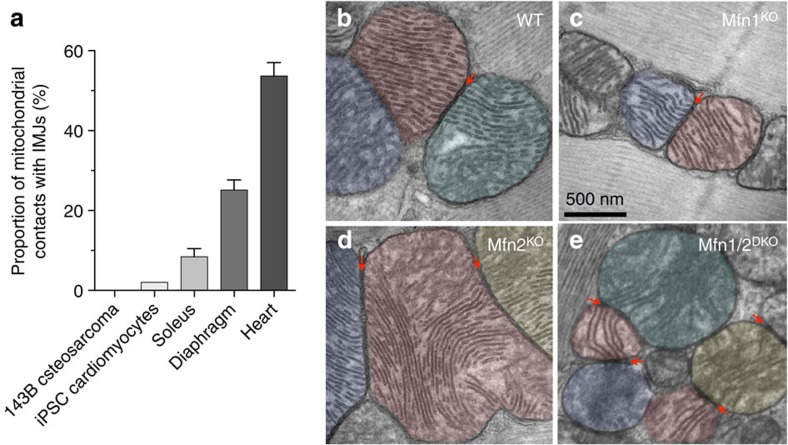
IMJs are physiologically regulated and do not require mitofusins. (**a**) Proportion of mitochondrial IMJs in various cells and tissues. (**b**) IMJs and cristae alignment occur in cardiomyocytes from wild type (WT), (**c**) Mfn1 knochout (*Mfn1*^KO^), (**d**) *Mfn2*^KO^ and (**e**) inducible *Mfn1*/*Mfn2*-double knockout (*Mfn1/2*^DKO^) mice.

Consistent with a physiological role of these interactions, the proportion of mitochondrial contacts harbouring enhanced electron density correlates with the cell’s reliance on oxidative phosphorylation for energy production. IMJ abundance progresses from non-existent in cultured 143B osteosarcoma cell lines, to <1% in induced pluripotent stem cells differentiated to the cardiomyocyte lineage *in vitro*, 8.4% in mouse skeletal muscle (soleus), 24.6% in the diaphragm (more oxidative than soleus) and 53.7% in the cardiac left ventricle (most oxidative; Fig. 4a).

Interestingly, mitochondrial IMJs are dynamically modulated in the absence of changes in mitochondrial content. We previously found that enhancing cellular energy demand in mouse skeletal muscle by a single bout of voluntary exercise increases mitochondrial IMJs by 1–2 fold, although the significance of this observation was not fully appreciated^25^. In contrast, reducing cellular energy demand in the diaphragm by suppressing contractile activity during mechanical ventilation tends to decrease IMJ abundance. In the heart, IMJs increase in proportion with pressure overload and vanish after prolonged cardiac arrest where mitochondria become depolarized^26^. IMJs also appear dynamically regulated in mitochondria isolated from their cellular context, on addition of electron donors to generate membrane potential^27^. Thus, IMJs are dynamic structures whose abundance and function are an intrinsic property of mitochondria physiologically regulated by their bioenergetic state.

IMJs could represent pre-fusion events halted by cytoplasmic factors in the state of inter-organelle tethering^28^. To determine whether this was the case, we tested whether IMJs and cristae alignment depend on mitofusins 1 and 2 (Mfn1 and Mfn2), the only two proteins currently known to mediate inter-mitochondrial tethering. In mice with heart-specific, germline deletion of either protein (Mfn1^KO^ and Mfn2 ^KO^), IMJs and cristae alignment still occur (Fig. 4b–d). In the cardiac-inducible Mfn1/Mfn2^DKO^ mice, which have almost complete loss of Mfn1 and Mfn2 after 3 weeks of induction^29^, trans-mitochondrial cristae alignment appears unaffected (Fig. 4e). Therefore, we conclude from this that mitofusins are not essential for the formation of the IMJ and trans-mitochondrial coordination of cristae.

### IMJs and cristae are induced by stably linking mitochondria

**Figure 5:**
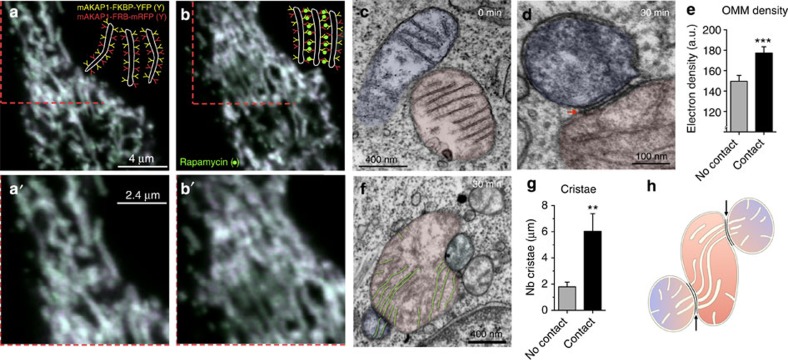
Mito–mito linker induces IMJs and coordination of cristae. (**a**,**a′**) Confocal imaging of RBL-2H3 cells transfected with mitochondria-targeted inducible linkers at 0 min and (**b**,**b′**) 30 min post-induction with rapamycin. (**c**,**d**) Electron micrographs from (**c**) non-induced and (**d**) 30 min post-induction of mitochondrial linkage, (**e**) showing increased electron density selectively at site of contacts (means±s.e.m., paired *T*-test, *n*=20 per group). (**f**,**g**) Quantification of cristae abundance at linker-induced contact sites compared with no contact mitochondrial surfaces (means±s.e.m., paired *T*-test, *n*=14 per group). (**h**) Theoretical model whereby cristae organization and density are regulated at IMJs, possibly enabling the equilibration of the membrane potential across physically tethered organelles. **P<0.01, ***P<0.001.

To determine whether the formation of IMJs and cristae alignment could be induced by the stable juxtaposition of mitochondrial OMMs, we used a novel drug-inducible system that physically links adjacent mitochondria together. RBL-2H3 cells were transiently transfected with two constructs, each encoding a component of the FKBP-FRB heterodimerization system linked to OMM-targeting domains. FKBP and FRB proteins project out of the OMM and include a rapamycin-binding site at their end. The addition of rapamycin binds the apposing OMM projections, physically tethering two mitochondria together. Confocal imaging of cells induced by rapamycin indicated stable docking of mitochondria within 30 min (Fig. 5a,a′).

Compared with non-induced cells, where physical contact between mitochondria is non-existent (Fig. 5c), induced tethering leads to increased OMM electron density along the length of inter-mitochondrial contacts, recapitulating IMJ structures (Fig. 5d,e). In addition, as in cardiomyocyte mitochondria, electron-dense sites of mitochondrial tethers contain significantly more cristae per membrane area than sites of mitochondria in contact with non-mitochondrial structures (Fig. 5f,g). These results further indicate the dynamic nature of these structures. Moreover, this demonstrates that both IMJs and cristae formation can be induced by stable juxtaposition of mitochondria, suggesting that physical contact of energized mitochondria can stimulate membrane interactions and cristae coordination (Fig. 5h).

## Discussion

Communication among biological organisms is a ubiquitous feature that pervades across the levels of organization—from cells to whole organisms. Recent studies suggest that subcellular information can be transferred between neighbouring mitochondria^7^,^8^,^9^,^10^,^11^. Our results now establish the existence of trans-mitochondrial cristae coordination at specialized IMJs. These features are conserved in various cell types across tissues and animal species, are resistant to genetic disruption of cristae morphology from altered oxidative phosphorylation, independent of mitofusins, yet inducible by physically and stably linking together adjacent mitochondria. In addition, IMJ numbers exist in proportion with cellular mitochondrial content and are dynamically regulated with energetic demand, being practically inexistent in cultured cells and most abundant in cardiomyocytes. Collectively, this data suggest that such specialized mitochondrial membrane structures exist to promote information transfer inside the cytoplasm of eukaryotic cells^30^.

Mammalian mitochondria evolved, in an endosymbiotic relationship with the eukaryotic cell, from their aerobic bacterial ancestors. The behaviour of bacteria is characterized by an extensive set of inter-cellular signalling mechanisms, such as the release of soluble molecules and physical membrane interactions (for example, for DNA transfer of antibiotic resistance genes). Collectively, organized processes of information transfer between bacteria enable ‘quorum sensing’—which trigger the coordination of gene expression patterns and synchronous behaviour among members of the bacterial colony^6^. A hallmark of bacterial quorum sensing is that bacteria–bacteria communication is preferentially induced at high population density^31^. Therefore, should inter-mitochondrial communication share an evolutionary-conserved functional role with bacterial processes, this fact would be compatible with the increased rate of IMJs in parallel with tissue mitochondrial density.

While the exact function of IMJs remains to be determined, one possibility is that the IMJs are ion channels allowing the electrochemical gradients of adjacent mitochondria to be coupled^7^,^10^,^32^, thereby enhancing the bioenergetic efficiency across functional clusters of mitochondria within the mitochondrial reticulum^13^,^33^,^34^. In this regard, the physical alignment of cristae across mitochondria would be relevant, since unlike other ions and molecules, the electrochemical potential generated within the cristae invaginations is distributed uniformly along their full length^35^. If the apposition of cristae junctions between two mitochondria at an IMJ resulted in a continuous inter-membrane space, then membrane potential could thus be transferred between organelles^7^. It may be that such putative channels are dynamically regulated, being only seldom captured in an open confirmation.

The extensive bending of cristae linked to IMJs implies regulation and functional significance. Natural and thermodynamically stable conformations of lipid bilayers are flat sheets. Relatively high degrees of membrane curvature can be induced and maintained by the presence of proteins within the membrane, such as the dimerized ATP synthase^36^. But in this particular case, incorporation of ATP synthase dimers in the IMM causes a circumferential curving of cristae that facilitates tubule formation (rather than sheets). This conformation is different than the longitudinal bending tubules or flat lipid sheets along the length of cristae, as it happens between two IMJs. The bending of cristae from one end to the other, a thermodynamically unfavourable conformation, must therefore involve the input of energy, implying regulation of cristae biogenesis, organization or both at IMJs^24^. This postulate is further supported by the increased number of cristae junctions at IMJs, both in the natural setting of muscle cells, and *in vitro* by the induced tethering of mitochondria. Furthermore, the functional association of IMJs and cristae is supported by the propensity of cristae to form right angles exclusively at IMJs, where cristae are rarely (<4% of all surveyed cases) oriented parallel to IMJs (see Fig. 3d).

Cristae architecture also directly influences respiratory properties and susceptibility to mitochondrial permeability transition leading to apoptotic signalling^37^. As a result, modulation of cristae organization by any factor, including mitochondrial interactions and trans-mitochondrial cristae coordination, could theoretically influence mitochondrial functions. In addition to the potential enhancement of intrinsic mitochondrial functions such as oxidative phosphorylation capacity and calcium handling, network connectivity provides physiological robustness ensuring energy supply is coordinated with cellular energy demand^38^. However, the possibility of a single fused mitochondrial reticulum would not be bioenergetically advantageous and could favour the spread of molecular mtDNA defects^39^. Therefore, the physiological coupling of distinct mitochondria by regulated membrane junctions may afford the advantage of maintaining separate organelles with relatively heterogeneous composition, while also benefiting from the synchronization of mitochondria’s energetic states within cells.

A few reasons may explain why trans-mitochondrial cristae coordination was not previously observed. First, only recently was the rapid exchange of electrochemical information between mitochondria unequivocally demonstrated^7^,^9^. This provided the necessary impetus for shifting high-resolution electron microscopy investigation of mitochondrial ultrastructure—from single mitochondria to mitochondrial dyads, with an emphasis on spatial correlations between organelles. Second, the alignment of cristae between mitochondria could appear as spurious events on single ultrathin sections of tissue in TEM. We found that electron tomography—the three-dimensional high-resolution imaging of thick tissue samples—was critical to provide compelling evidence for the coordination of multiple cristae through the thickness of multiple mitochondria (for example, Supplementary Video 1). The use of tomography is also primordial to quantify the number of cristae junctions at IMJs and non-IMJ contacts. Finally, cristae coordination at IMJs, or the lack thereof, was made particularly obvious on examination of animal models with naturally disorganized and dysmorphic cristae (see Fig. 3a). Thus, the combination of these approaches, along with quantitative assessment of cristae angles and inter-organelle coordination, were critical to document these structures.

In summary, our morphological analysis of mitochondrial interactions reveals that adjacent mitochondria can interact through specialized, regulated IMJ sites, where cristae membranes become organized into coordinated pairs across organelles. These conserved features reveal an unsuspected level of interaction between mitochondria and may provide a structural basis for observed rapid events of electrochemical inter-mitochondrial communication. At present, incompatible methodology between that required to resolve these ultrastructural features and the experimental conditions necessary for effective immunolabelling preclude clarification of potential IMJ constituents. Future studies will be required to uncover the full physiological significance of these structures, as well as their molecular composition and the mechanisms that orchestrate their regulation.

## Methods

### Animals and tissue collection

All protocols were approved by the Institutional Animal Care and Use Committees from the Children’s Hospital of Philadelphia, Thomas Jefferson University or the University of Washington at St Louis. For analyses of mouse tissues, 8–12-month old C57BL/6EiJ mice were used. Animals were euthanized by cervical dislocation, the left myocardium was immediately dissected into <0.5 mm thick slices and fixed as described below. For the skeletal muscle, the soleus was cut longitudinally along the midline and fixed under identical conditions.

Transgenic mitochondrial mutant mice with dysmorphic cristae were created by combining specific pathogenic alterations in both the mtDNA and nuclear DNA. Briefly, female mice homoplasmic for a mtDNA point mutation at nucleotide G13997A in the *ND6* gene causing the amino-acid substitution P25L^23^, were crossed onto a nuclear DNA background (Fig. 3e,f) lacking the *Ant1*^−/−^ (ref. 22). The resulting double mutant mice presented with severely disrupted cristae, consistent with a role of the OXPHOS components and function in determining cristae shape^36^.

Mice lacking Mfn1 and Mfn2 were generated using a full-length MYH6 driven Cre recombinase to obtain postnatal Mfn2 cardiac-specific deficiency^40^ (Fig. 4b). To circumvent the embryonic lethality caused by ablation of both mitofusins, cardiac-specific Mfn1/Mfn2^DKO^ mice were generated using a tamoxifen-inducible modified oestrogen receptor cardiac-specific *MYH6*–Cre system, resulting in 80–85% loss of Mfn1 and Mfn2 after 3 weeks^29^.

### Preparation of samples for TEM

Samples were immediately fixed by immersion in a 2% glutaraldehyde solution in 0.1 M cacodylate, buffer (pH 7.4)^41^. After subsequent buffer washes, samples were post-fixed in 2.0% osmium tetroxide for 1 h at room temperature and rinsed in distilled H_2_O before the in-bloc staining with 2% uranyl acetate. After dehydration through a graded ethanol series, each sample was infiltrated and embedded in EMbed-812 (Electron Microscopy Sciences, Fort Washington, PA). To verify orientation and section quality, 1-μm-thick sections were cut and stained with 1% toluidine blue. Thin sections (90 μm) were mounted on filmed copper grids and stained with uranyl acetate and lead citrate and examined on a JEOL 1010 electron microscope fitted with a Hamamatsu digital camera and AMT Advantage image capture software.

We found that glutaraldehyde-only fixation is primordial for preservation of electron density in IMJs, whereas paraformaldehyde is not optimal. Post-staining of ultrathin sections (with uranyl acetate and lead citrate) was not necessary to visualize electron-dense IMJs, which were visible from en-block-only osmium tetroxide-stained specimens. In addition to published images from laboratories worldwide where IMJs and cristae features can be observed (see Supplementary Table S1 and associated images), our observations of IMJs in mouse heart and skeletal muscle were reproducible in samples collected in seven different laboratories across eight strains of mice, with tissues processed and imaged in five different imaging facilities/laboratories.

For tomography, ~150–300-μm-thick sections were imaged by dual axis image tilt series from −60° to +60° in 1.5 degree increments on a FEI Tecnai 12 microscope equipped with a Gatan US-1000 camera at × 6,500–12,000 magnification using SerialEM^42^. Tomographic volumes were reconsructured within the etomo software package^43^. Analysis and video synthesis were performed using 3Dmod (IMOD 4.7, Boulder Laboratory for 3-D Electron Microscopy of Cells) and Image J (NIH, version 1.47v).

### Membrane electron density

Electron micrographs from the heart at × ~30,000–100,000 indirect magnification (pixel length: 1.31–2.20 nm, respectively) were analysed using Image J. Mitochondrial membranes on calibrated 8-bit images, with each pixel representing ~1.8 nm, were manually traced. Area was converted to line, a pixel intensity plot generated, and data exported to Microsoft Excel. For each mitochondrion analysed (*n*=20), intensity was measured for the surrounding cytoplasm, matrix space, OMM and IMM (without contact), cristae, and OMM at IMJs. Values were normalized to cytoplasm intensity to standardize overall image brightness. Final membrane intensity values (Fig. 1c,d) were obtained by taking the inverse of averaged intensity values minus background. Whereas most IMJs could be resolved at a single-membrane level, at some IMJ OMM membranes could be distinguished when lipid bilayer leaflets were not precisely perpendicular to the sectioning/imaging plane.

### Cristae density and alignment

To quantify the surface density of cristae (number of cristae per membrane surface area) at IMJs and non-IMJ contacts, three-dimensional tomograms were manually analysed. Junctions between cristae and the IMM outer boundary membrane (cristae junctions) could thus be accurately determined. In total, 510 cristae junctions were analysed, derived from four complete tomographic reconstructions containing 14 interacting mitochondria. On average, tomograms were 221-nm thick. In each reconstruction plane, cristae junctions were individually numbered and traced until a fenestration was encountered, thus revealing discontinuity (Fig. 2a, Supplementary Fig. 2). For each mitochondrion (using paired analysis) cristae density normalized to μm^2^ of outer boundary membrane was compared between areas of the mitochondrion forming IMJs with other mitochondria, and areas of the same mitochondrion in contact with non-mitochondrial structures (i.e., myofilaments) (Fig. 2b).

To evaluate whether cristae between adjacent mitochondria were physically coordinated, cristae angle within the picture frame were measured in Image J on images at × 12,000 indirect magnification (pixel length: 11.02 nm). In total, 151 (IMJ) and 83 (non-IMJ contact) pairs of adjacent mitochondria were analysed, for a total of 302 and 166 mitochondria. If cristae were perfectly aligned, the resulting angle between them resulted in a 0° angle, whereas complete non-alignment resulted in a 90° incident angle. In addition, cristae orientation relative to the IMJ contact site tangent was determined for each mitochondrion, and angles analysed.

### Mito–mito linker experiments

To test whether physically tethering mitochondria to one another could increase OMM electron density and influence cristae organization, we used an engineered synthetic mito–mito linker system in mammalian cells. RBL-2H3 rat cells (ATCC) were transiently co-transfected with two constructs consisting of (1) OMM-targeting sequence (mAKAP1;34–63), linker domain FKBP12 and fluorophore YFP (mAKAP1-FKBP12-YFP) and (2) mAKAP1(34–63) linker domain FRB and fluorophore mRFP (mAKAP1-FRB-mRFP). mAKAP1-FRB-mRFP was created by replacing the N-terminal-targeting sequence of Lyn in the PM-FRB-mRFP construct^44^ with the coding sequence of mAKAP 34–63 resulting an identical linker (DPTRSANSGAGAGAGAILSR) with the one between AKAP and FKBP. To induce heterodimerization of the FKBP/FRB linker pair, 100 nM rapamycin was applied^44^. As a result, pairs of mitochondria become stably tethered after 30 min (see Fig. 5).

Cells were cultured and transiently transfected with plasmid DNA by electroporation using 4.5 × 10^6^ cells+20 μg of each complementary DNA in 250 μl medium. Electroporation was performed with a BTX-830 square-pulse generator in a 4-mm gap cuvette using a single 250-V 13-ms pulse^15^. Transfection efficiency was ~50%. Ten to fifteen minutes before induction of linkage, cells were transferred to a Ca^2+^-free media. Induction was initiated by incubation with rapamycin. Live cell imaging was conducted on a Zeiss LSM 780 inverted confocal microscope system (× 63, 1.4 numerical aperture oil objective, 70 nm pixel size). Cells were then fixed in a 2% glutaraldehyde and 0.5% tannic acid solution in 0.1 M cacodylate, buffer (pH 7.4) for 15 min. Fixed cells were then liberated from the dish using a cell scraper and further processed for TEM embedding as described earlier^45^. Membrane electron density and cristae abundance per surface area were performed exactly as for cardiomyocytes. Cultured cells possessed too few cristae to analyse cristae orientation and alignment.

### Statistical analyses

Student’s *t*-tests or Mann–Whitney, one-way analysis of variance, 99% confidence intervals and *χ*^2^ tests were performed in Prism 6.

## Additional information

**How to cite this article:** Picard, M. *et al*. Trans-mitochondrial coordination of cristae at regulated membrane junctions. *Nat. Commun.* 6:6259 doi: 10.1038/ncomms7259 (2015).

## Supplementary Material

Supplementary Figures, Supplementary Table, Supplementary References Supplementary Figures 1-3, Supplementary Table 1, Supplementary Reference

Supplementary Movie 1 Animated Z-series of electron tomogram showing trans-mitochondrial cristae coordination (0:16 min). Section of heart cardiomyocyte (thickness: 197nm) in mice with naturally dysmorphic cristae (Ant1-/-;ND6P25L), imaged by dual axis electron tomography (see methods for details). The central mitochondrion makes contact with four surrounding mitochondria. At these sites of contact marking the top, left and bottom segments of the central mitochondrion, cristae form organized parallel arrays to each other. They also exhibit few fenestrations. Only at the right margin of the central mitochondrion, where it contacts non-mitochondrial cellular components (i.e., myofibrils and sarcoplasmic reticulum), are cristae disorganized. At inter-mitochondrial junctions, cristae of the central mitochondrion are aligned in space with those of neighbouring mitochondria. This can be noticed by their physical apposition throughout the three-dimensional space as the volume is visualized. Note how the near parallel transmitochondrial alignment entails cristae curvature. In so doing, cristae physically extend between two inter-mitochondrial junctions.
