# Supplementary material for: Trans-mitochondrial coordination of cristae at regulated membrane junctions
Source: Nat Commun. 2015 Feb 17;6:6259. doi: 10.1038/ncomms7259 (PMC4332397; doi:10.1038/ncomms7259)
Supplement: Supplementary Figures, Supplementary Table, Supplementary References — Supplementary Figures 1-3, Supplementary Table 1, Supplementary Reference [file ncomms7259-s1.pdf]

Supplementary Figure 1

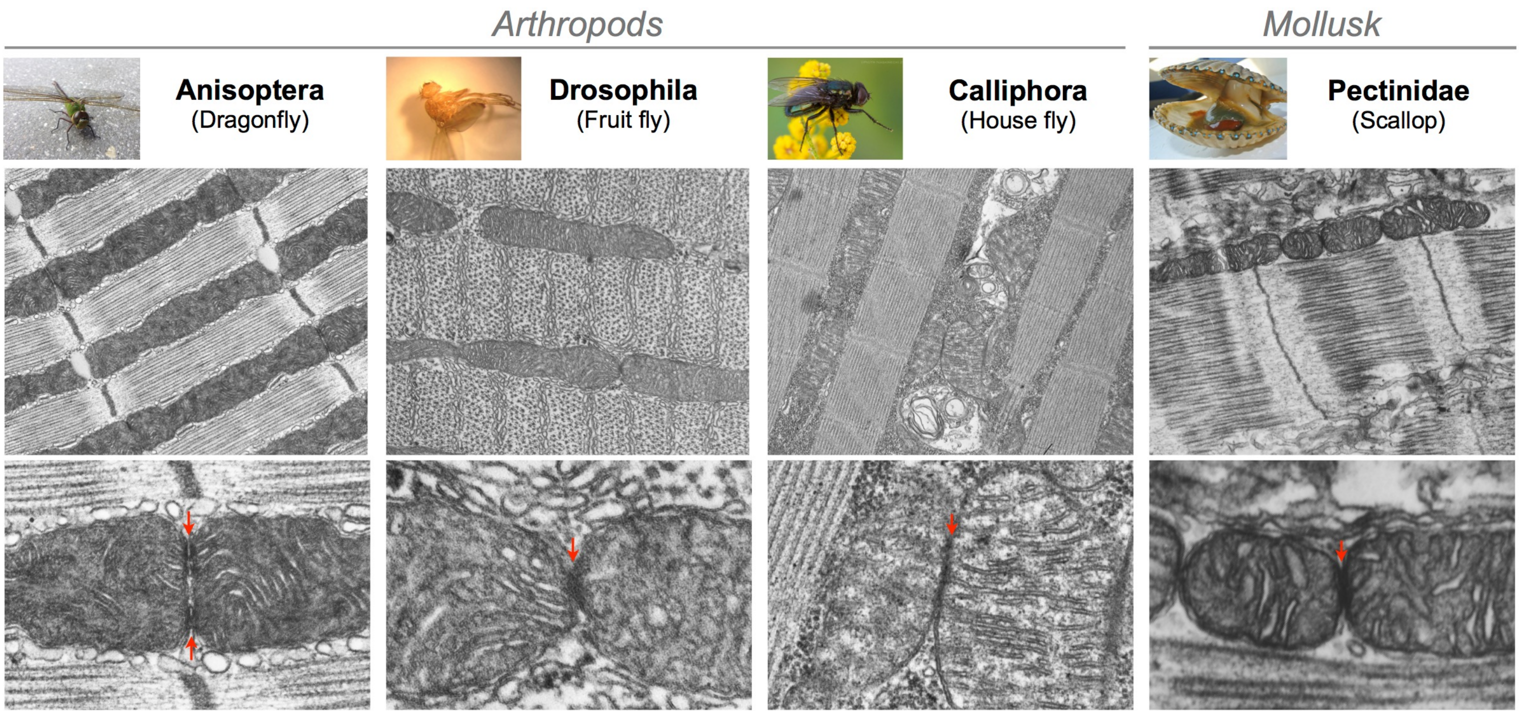

Electron-dense inter-mitochondrial junctions (IMJs) and associated cristae organization are evolutionary conserved features of mitochondria-mitochondria contacts. Shown are electron micrographs from three arthropod species and one mollusk muscle at low (top) and higher (bottom) magnification. Samples were fixed by immersion with glutaraldehyde, as described in the methods. Images were kindly provided by Dr. Clara Franzini-Armstrong.

Supplementary Figure 2

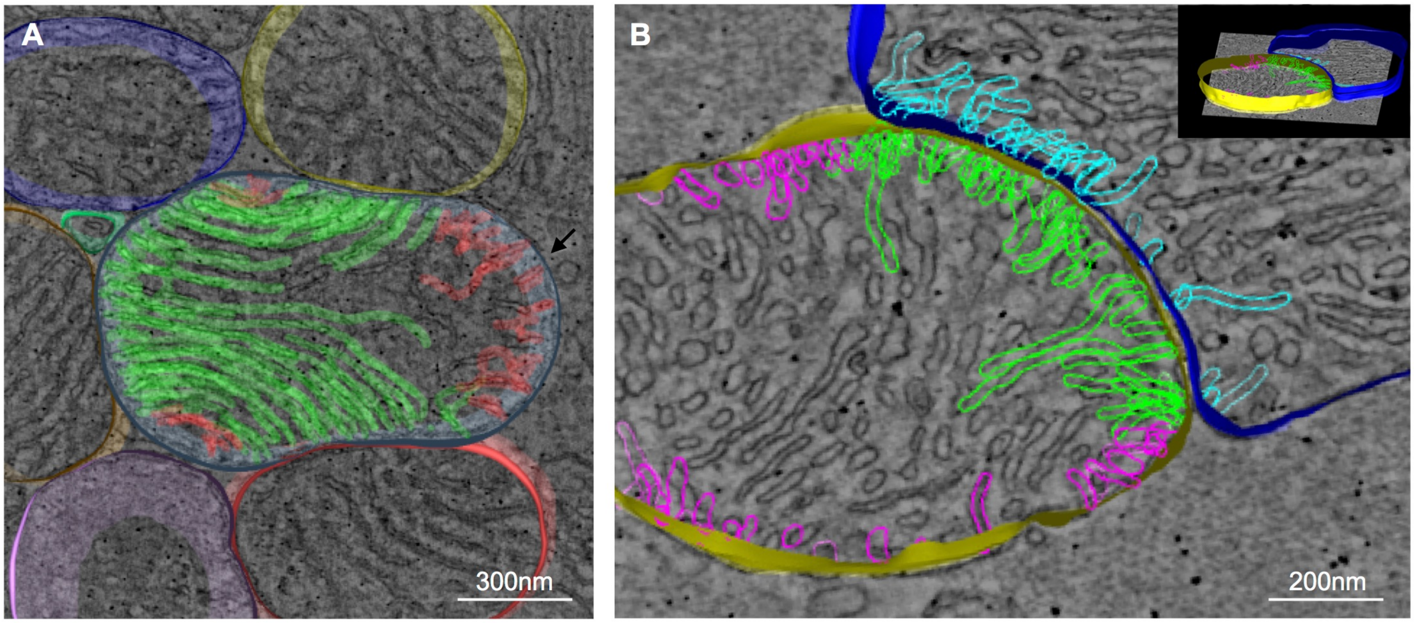

Electron microscopy tomographic reconstructions show cristae number, length and organization. (**A**) Electron tomogram (thickness = 197nm) of mouse heart cardiomyocyte mitochondria, generated as described in materials and methods. Mitochondrial membranes are outlined in colours. Profiles of uninterrupted cristae originating from IMJs (green) and non-IMJ membrane (red), showing both cristae density and length. Note the discontinuous and heterogenous orientation of cristae originating from areas of mitochondria facing myofibrils (arrow), compared to areas of the same central mitochondrion in contact with other mitochondria. (**B**) Electron tomogram (thickness: 157nm) of mouse skeletal muscle generated as in A to quantify the number of cristae junctions.

Supplementary Figure 3

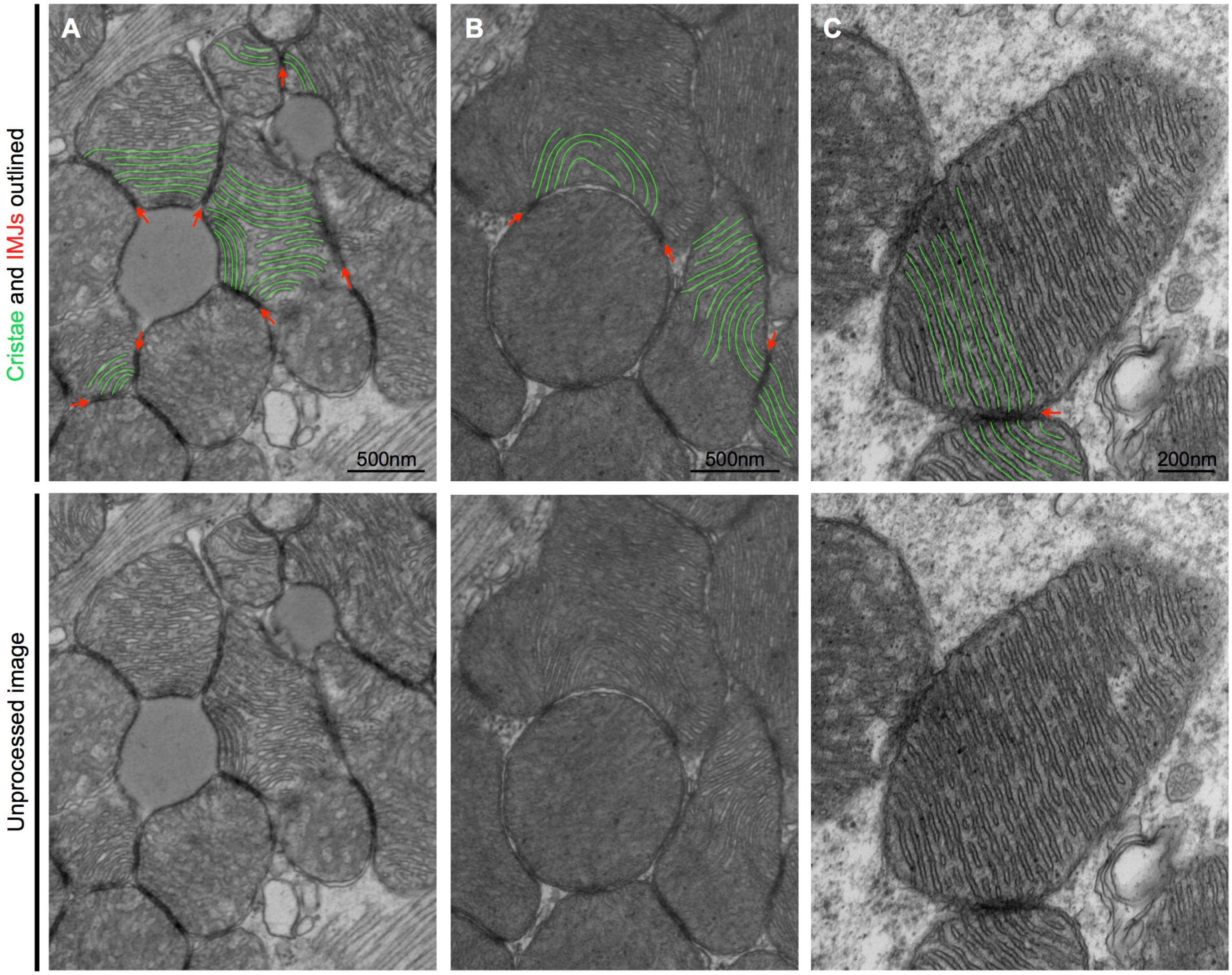

Electron micrographs showing cristae organization and their relation to IMJs. (**A, B**) Mouse heart cardiomyocyte and (**C**) skeletal muscle (soleus) mitochondria, with selected cristae outlined in green. Shown are both raw images (bottom) and with cristae tracing outlined (top). Image in B kindly provided by Drs. Yan Burelle and Virgilio Cadete.

Supplementary Table 1

Species	Tissue	Cell type/sub-region	Reference
Mouse	Heart	Cardiomyocyte, left myocardium	1
	Muscle	Soleus	2, 3
Rat	Heart	Cardiomyocytes	4
	Heart	Cultured primary cardiomyocytes	5
	Diaphragm	Skeletal muscle fibers	6
Human	Eye	Retina	7
	Tumor	Glioma	8
Lamprey	Brain	Presynaptic terminal (tonic synapses > phasic synapses)	9
Bat	Fat	Brown adipose tissue	7
Squirrel	Brain	Hippocampus, CA1 neurons	10
Hamster	Adrenal gland	Cortex, zona fasciculata	7
Frog	Heart	Cardiomyocytes	11

Non-comprehensive list of published sources where inter-mitochondrial junctions (IMJs) can be observed.

Supplementary References

1. Hollander JM, Thapa D, Shepherd DL. Physiological and Structural Differences in Spatially-Distinct Subpopulations of Cardiac Mitochondria: Influence of Pathologies. *Am J Physiol Heart Circ Physiol*, (2014).

2. Picard M, White K, Turnbull DM. Mitochondrial morphology, topology, and membrane interactions in skeletal muscle: a quantitative three-dimensional electron microscopy study. *J Appl Physiol* **114**, 161-171 (2013).

3. Picard M*, et al.* Acute exercise remodels mitochondrial membrane interactions in mouse skeletal muscle. *J Appl Physiol* **115**, 1562-1571 (2013).

4. Bakeeva LE, Chentsov Yu S, Skulachev VP. Intermitochondrial contacts in myocardiocytes. *J Mol Cell Cardiol* **15**, 413-420 (1983).

5. Amchenkova AA, Bakeeva LE, Chentsov YS, Skulachev VP, Zorov DB. Coupling membranes as energy-transmitting cables. I. Filamentous mitochondria in fibroblasts and mitochondrial clusters in cardiomyocytes. *J Cell Biol* **107**, 481-495 (1988).

6. Bakeeva LE, Chentsov Yu S, Skulachev VP. Mitochondrial framework (reticulum mitochondriale) in rat diaphragm muscle. *Biochim Biophys Acta* **501**, 349-369 (1978).

7. Fawcett DW. The Cell, Chapter 7: Mitochondria. In: *The Cell* (ed^(eds). W. B. Saunders Company (1981).

8. Arismendi-Morillo G. Electron microscopy morphology of the mitochondrial network in gliomas and their vascular microenvironment. *Biochim Biophys Acta* **1807**, 602-608 (2011).

9. Brodin L, Bakeeva L, Shupliakov O. Presynaptic mitochondria and the temporal pattern of neurotransmitter release. *Philos Trans R Soc Lond B Biol Sci* **354**, 365-372 (1999).

10. Popov V, Medvedev NI, Davies HA, Stewart MG. Mitochondria form a filamentous reticular network in hippocampal dendrites but are present as discrete bodies in axons: a three-dimensional ultrastructural study. *J Comp Neurol* **492**, 50-65 (2005).

11. Duvert M, Mazat JP, Barets AL. Intermitochondrial junctions in the heart of the frog, Rana esculenta. A thin-section and freeze-fracture study. *Cell Tissue Res* **241**, 129-137 (1985).
